# An Autopsy Case of Pulmonary Tumor Emboli Due to Metastatic Squamous Cell Carcinoma

**DOI:** 10.7759/cureus.19506

**Published:** 2021-11-12

**Authors:** Mohammed Alsaggaf, Amandeep S Bawa, Rahul Khosla

**Affiliations:** 1 Pulmonary and Critical Care Medicine, George Washington University Hospital, Washington DC, USA; 2 Pulmonary and Critical Care Medicine, Veterans Affairs Medical Center, Washington DC, USA

**Keywords:** metastasis, embolism, squamous cell carcinoma of unknown primary, shortness of breath, pulmonary tumor embolism

## Abstract

A 74-year-old man with chronic obstructive pulmonary disease on home oxygen and coronary artery disease was transferred from an outside facility to obtain an inguinal lymph node biopsy to rule out malignancy. He underwent an uncomplicated procedure and was discharged the same day. While waiting for transportation, he had sudden-onset dyspnea and collapsed. After resuscitation, patient had return of spontaneous circulation and was admitted but was provided comfort care and soon expired. Autopsy showed metastatic squamous cell carcinoma with multiple bilateral tumor emboli. Pulmonary tumor embolism is a rare cause of dyspnea in cancer population. Most of the cases are diagnosed with autopsy after sudden death; however, few cases have been reported antemortem. Tumor embolism is rare and difficult to diagnose without an autopsy with a poor outcome.

## Introduction

Shortness of breath among cancer patients can be due to several causes, such as infection, metastasis, thromboembolism, and other cardiopulmonary diseases [1]. Pulmonary tumor embolism syndrome (also known as pulmonary tumor embolic microangiopathy “PTEM”) is a rare cause of dyspnea in cancer population [2]. We present a case of pulmonary tumor embolism. 

## Case presentation

**Figure 1:**
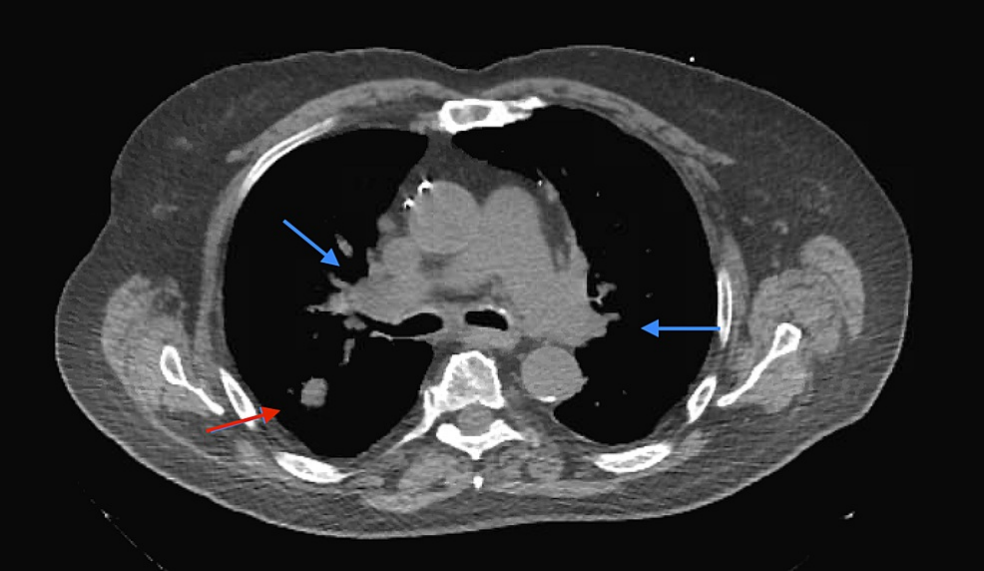
CT chest (mediastinal window) Bilateral mediastinal lymphadenopathy (blue arrows) + right upper lobe nodule (red arrow)

**Figure 2:**
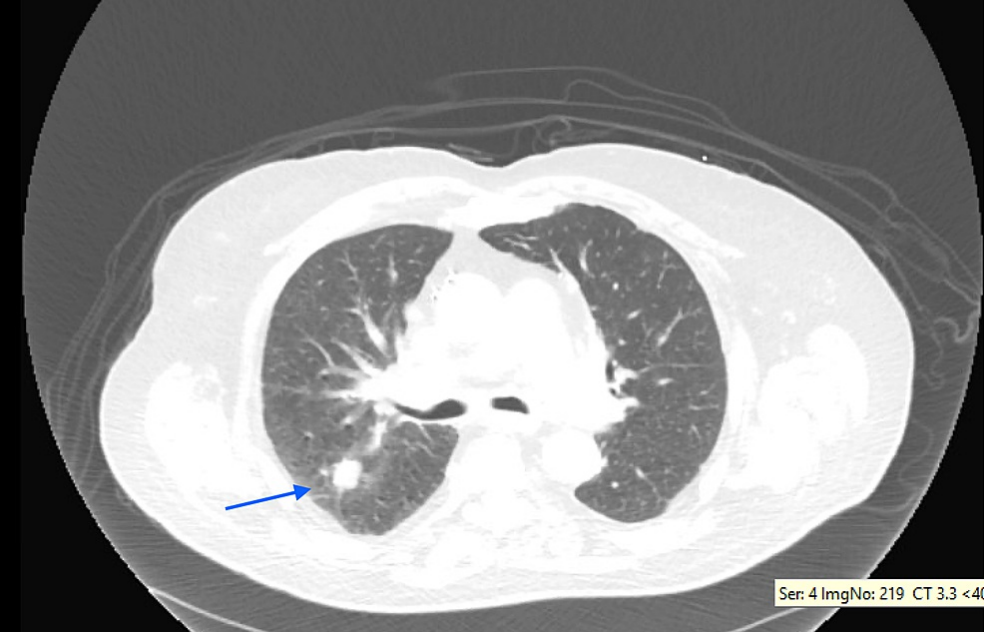
CT chest (lung window) Right upper lobe nodule (blue arrow)

**Figure 3:**
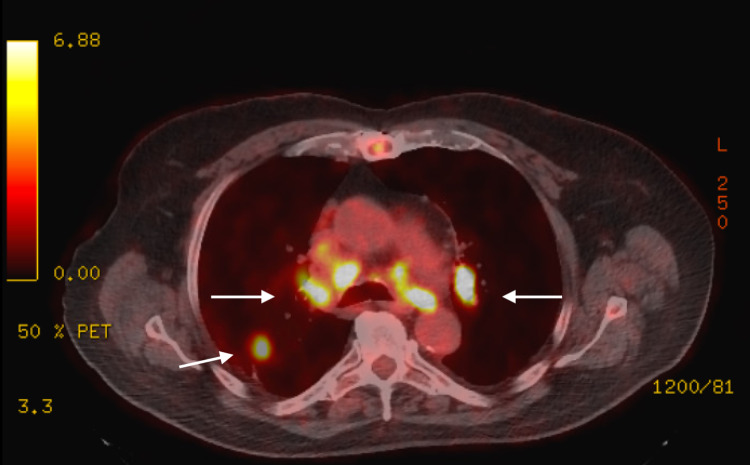
PET scan Intense FDG uptake among right upper lobe nodule and bilateral mediastinal lymph nodes (white arrows) PET, positron emission tomography; FDG, fluorodeoxyglucose

**Figure 4:**
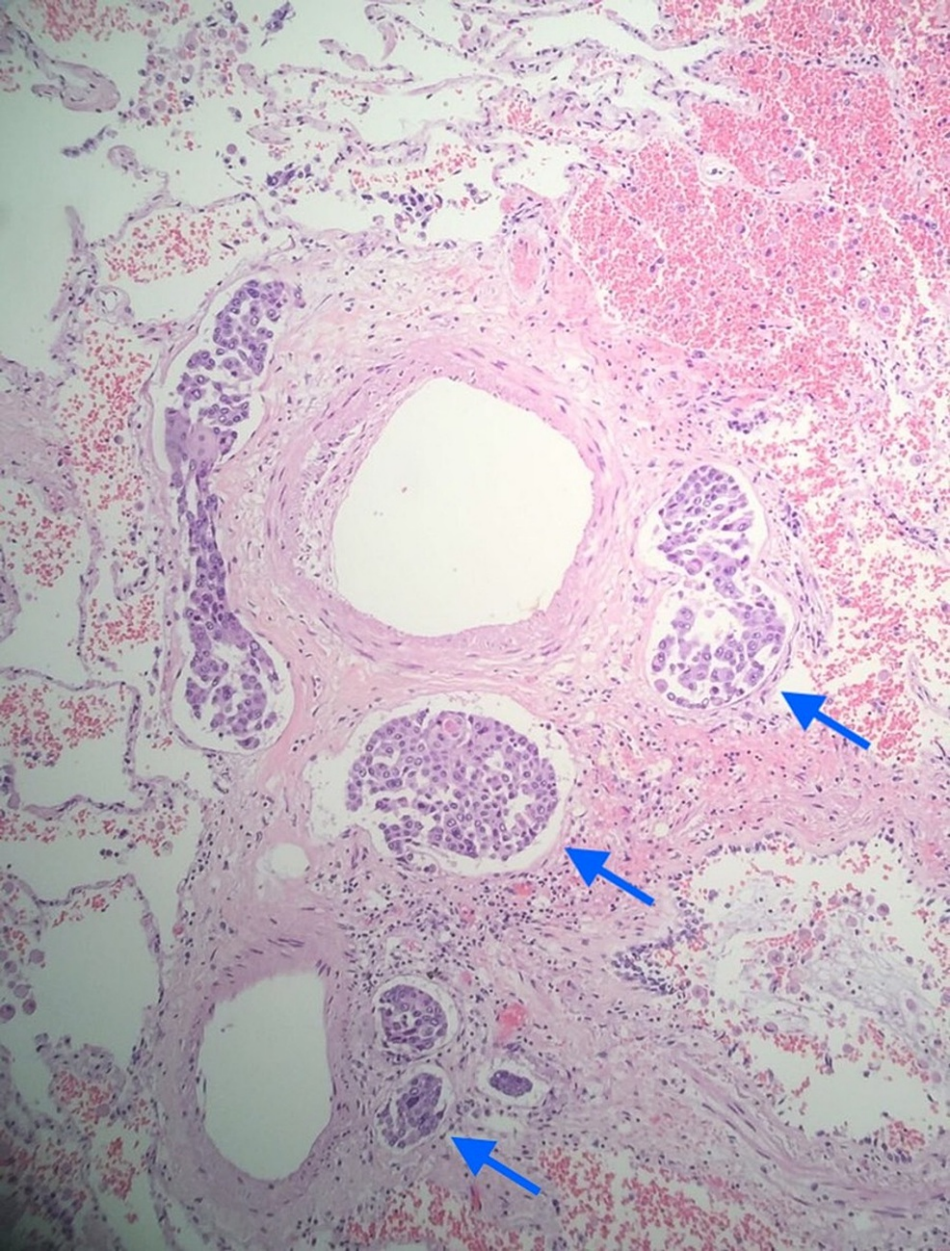
Lung autopsy Occlusion of small muscular pulmonary artery by tumor cells (blue arrows)

We present the case of a 74-year-old male with a history of severe chronic obstructive pulmonary disease on home oxygen, coronary artery disease status post coronary artery bypass grafting, and hypertension who was transferred from an outside hospital for lymph node biopsy. A chest CT scan showed scattered bilateral pulmonary nodules and diffuse lymphadenopathy (Figures 1, 2). A computed tomography-positron emission tomography (CT-PET) scan showed significant uptake in some of the lung nodules and many lymph nodes (Figures 1-3). The patient underwent an uncomplicated left groin lymph node biopsy and was discharged in a stable condition post-procedure. While waiting for his transportation, he had sudden-onset dyspnea and collapsed. After 40 minutes of cardiopulmonary resuscitation (CPR) for a non-shockable rhythm, patient achieved return of spontaneous circulation. An echocardiogram during CPR showed right heart strain with questionable inferior vena cava (IVC) clot; hence, tissue plasminogen activator was given for possible massive pulmonary embolism. Patient was admitted to the medical intensive care unit, but, after another cardiac arrest, his family provided him comfort care and he soon expired. Autopsy showed metastatic squamous cell carcinoma involving both lungs with extensive lympho-vascular invasion and tumor thrombus formation on histologic sections (Figure 4). No thrombus was grossly visualized in the bilateral pulmonary arteries and IVC. 

## Discussion

Arterial tumor emboli (ATE) are a fatal but rare complication of metastatic diseases. In 1975, Kane and Hawkins described few cases of unexplained dyspnea who were diagnosed to have multiple microscopic tumor emboli [1]. On autopsy, 3-26% patients have been reported to have PTEM [1]. ATE can be large enough to cause ischemia with organ infarction or can be very small and unrecognized until they progress and cause symptoms. Primary and secondary pulmonary malignancies are considered among the most common risk factors. ATE mostly occur perioperatively or immediately postoperatively. However, few spontaneous cases have been reported [2]. 

Pulmonary tumor cells in vasculature can be seen in four forms: 1) large, 2) proximal, 3) generalized lymphatic dissemination (lymphangitic carcinomatosis); and 4) pure microvascular disease or combination of them [1]. In regard to the mechanism of arterial embolization of pulmonary malignancies, it is acknowledged that pulmonary veins are invaded by tumor nests and then they are released into systemic circulation [2,3]. Another significant alleged risk factor is manipulation of an invasive tumor by surgical intervention, respiratory movements, or endothelial injury. Symptoms of organ infarction or ischemia are similar to atherosclerotic emboli. Surgical manipulation of malignancies such as lung cancer, sarcoma, germ cell tumor, and thyroid cancer can cause ATE [3]. Common locations of tumor emboli include aorta, intra-cerebral arteries, common femoral arteries, and iliac arteries [3]. In our case, tumor manipulation after inguinal lymph node biopsy could be a reason for the ATE causing cardiac arrest.

Patients with tumor emboli typically have a negative chest radiograph and CT scan. Tumor emboli and malignant cells can be seen in blood obtained from wedged pulmonary artery catheter [4,5]. In a case report of breast cancer with lung metastasis by Kayatani et al., PTEM was identified by transbronchial lung biopsy. In a clinical analysis of 30 autopsy cases by Uruga, it was found the survival after onset of dyspnea was on an average 16.2 days and after initiation of oxygen it was an average of 9 days [6]. Treatment options include targeted chemotherapy (e.g. breast cancer with lung metastases and renal cell cancer) [7] or surgical resection of the primary tumor (atrial myxoma) [8]. 

## Conclusions

Pulmonary tumor embolism is an uncommon complication of malignancy. It should be considered as a differential diagnosis in patients with malignancy or metastasis who develop symptoms and signs of pulmonary embolism. 
